# Supplementary figures and images for: Intron Regions as Genetic Markers for Population Genetic Investigations of Opisthorchis viverrini sensu lato and Clonorchis sinensis
Source: Animals (Basel). 2023 Oct 13;13(20):3200. doi: 10.3390/ani13203200 (PMC10603628; doi:10.3390/ani13203200)

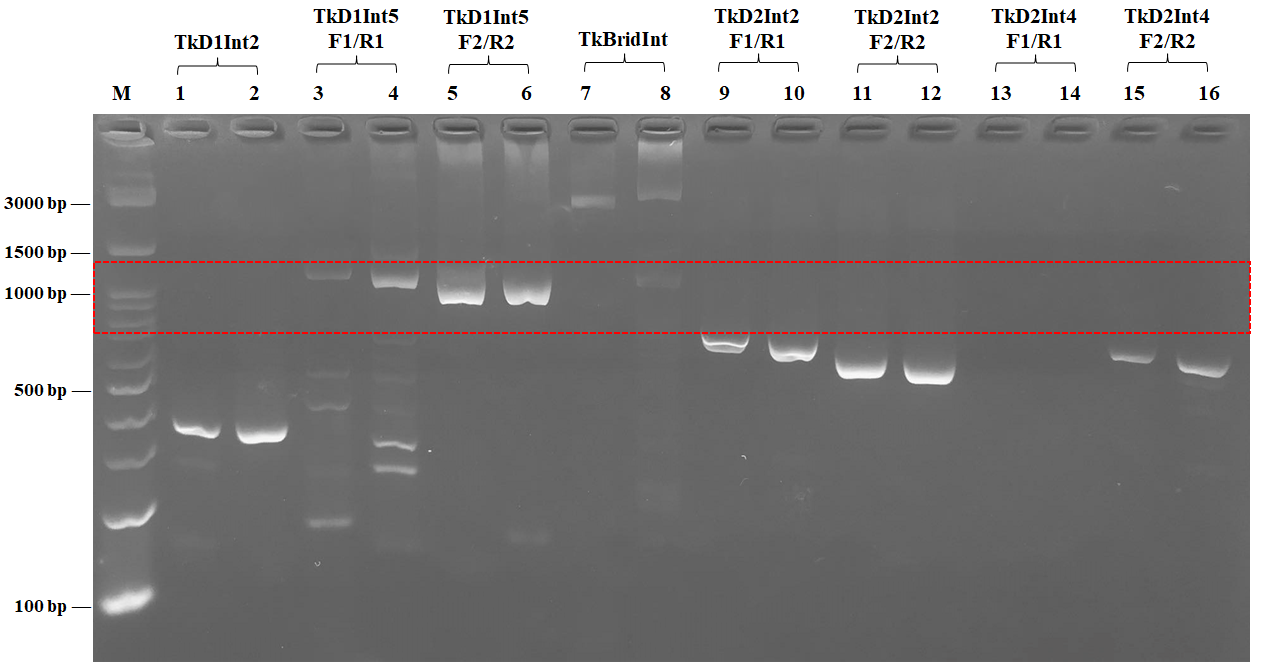

Supplement: Supplementary file 1 [file animals-13-03200-s001.zip › Figure S1.tif]

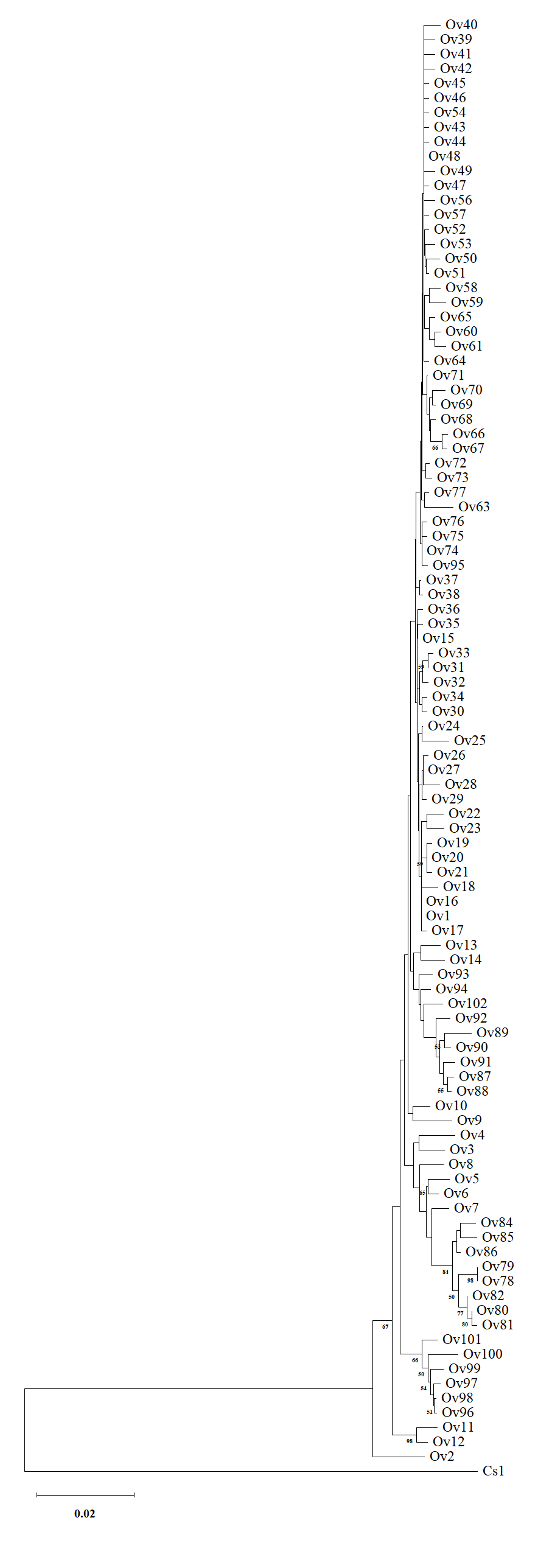

Supplement: Supplementary file 1 [file animals-13-03200-s001.zip › Figure S2.tif]

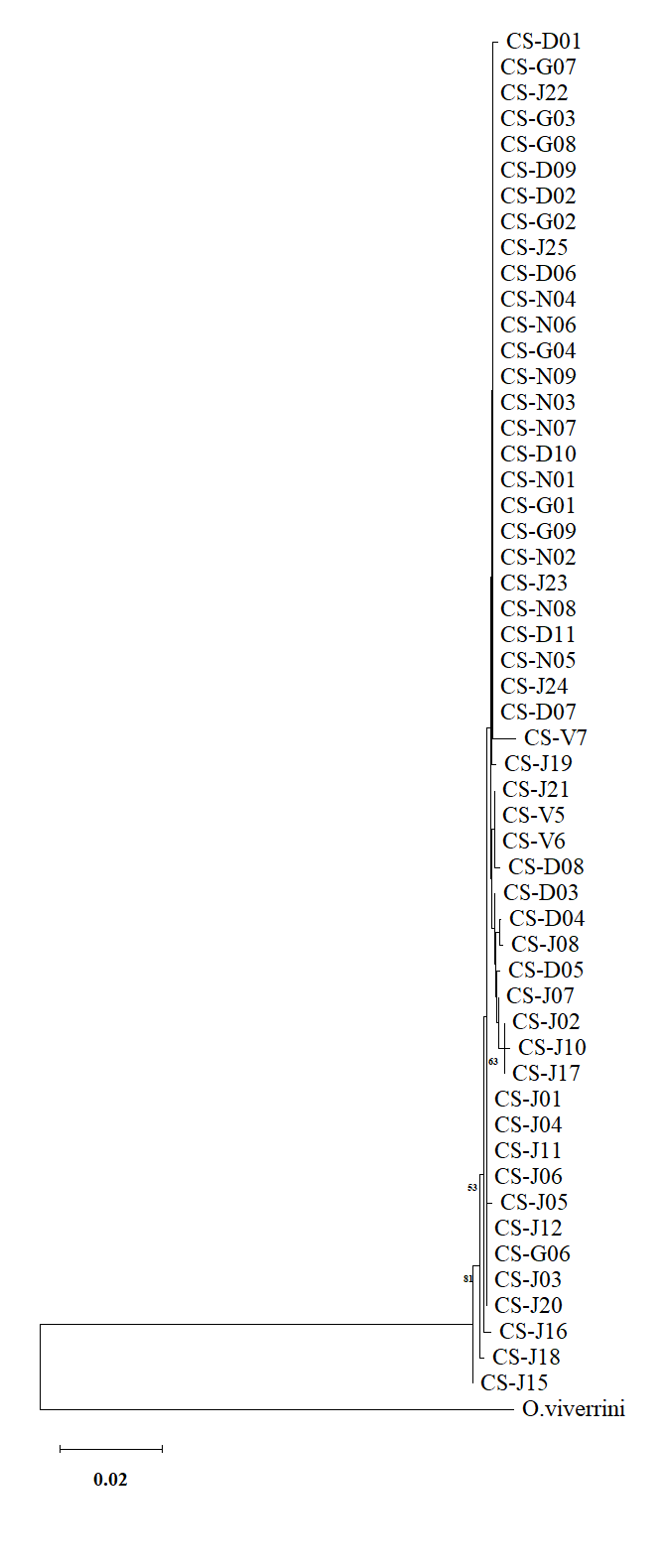

Supplement: Supplementary file 1 [file animals-13-03200-s001.zip › Figure S3.tif]
